# 

**Published:** 1821-08

**Authors:** 


THE LONDON
### Medical and Physical Journal.

2 OF VOL. XLVI.] AUGUST, 1821. [NO. 270.

### NOTICE.

*Another of the series of* PRŒMIA *to the several volumes of this Journal, (which commenced with that to the forty-third volume,) comprising a History of the Progress of Medicine and its auxiliary Sciences for the half-year immediately previous to the period of their production, respectively, was published on the last day of July. One of the especial intentions of those* Prœmia, *is to present a comprehensive view of the state and progress of Medicine throughout Europe generally, and in the United States of America; an object that cannot be effected in the regular monthly Numbers of the Journal, because of the small extent of space which can be there appropriated to this purpose.*

### MONTHLY CATALOGUE OF MEDICAL BOOKS.

Observations on certain Affections of the Head, commonly called Head-aches; with a view to their more complete Elucidation and Cure; together with some brief Remarks on Digestion and Indigestion. By James Farmer, Member of the Royal College of Surgeons in London, and Licentiate of Midwifery of the Royal College of Physicians, Dublin. 18mo. 2s.

The Quarterly Journal of Foreign Medicine and Surgery, and of the Sciences connected with them. No. XI. 8vo. 3s. 6d.

Medico-Chirurgical Transactions; published by the Medico-Chirurgical Society of London. Vol. XI. Part II. 9s. boards.

A Practical Treatise on the Inflammatory, Organic, and Sympathetic Diseases of the Heart; also on Malformations of the Heart, Aneurism of the Aorta, Pulsation in Epigastrio, &c. &c. By Henry Reeder, M.D. Member of the Medical and Chirurgical Society of London, and of the Royal Medical Society of Edinburgh. 8vo. 9s. 6d.

Researches into the Laws and Phenomena of Pestilence; including a Medical Sketch and Review of the Plague of London in 1665, and Remarks on Quarantine. With an Appendix, containing Extracts and Observations relative to the Plagues of Morocco, Malta, Noya, and Corfu; being the Subject of the Anniversary Oration delivered before the Medical Society of London, in the Spring of 1820, and published by its request. By Thomas Hancock, M.D. Licentiate of the Royal College of Physicians, and one of the Physicians to the City and Finsbury Dispensaries. 8vo. 8s.

A Practical Essay on Ringworm of the Scalp, Scalled Head, and other Species of Porrigo. By Samuel Plumbe, Member of the Royal College of Surgeons.

Elements of Medical Logic; illustrated by practical Proofs and Examples. The second Edition, with large Additions, particularly in the practical Part. By Sir Gilbert Blane, Bart. Physician to the King.

The Art of invigorating and prolonging Life, by Food, Clothes, Air, Exercise, Wine, Sleep, &c.; and peptic Precepts, pointing out agreeable and effectual Methods to prevent and relieve Indigestion, and to regulate and strengthen the Actions of the Bowels. By the Author of the Cook's Oracle.

The Principles of Forensic Medicine, systematically arranged, and applied to British Practice. By John Gordon Smith, M.D.

[HIGHLEY AND SON, FLEET-STREET.]

